# Willingness of nursing undergraduates to work in long-term care facilities: A qualitative study

**DOI:** 10.1371/journal.pone.0357086

**Published:** 2026-08-28

**Authors:** Xiufang Hou, Yanglan Wang, Fangfang Wang, Qiuping Li

**Affiliations:** 1 School of Nursing, Wannan Medical University, Wuhu, Anhui, China; 2 Nursing Department, Capital Medical University Xuanwu Hospital, Beijing, China; The University of York, UNITED KINGDOM OF GREAT BRITAIN AND NORTHERN IRELAND

## Abstract

**Purpose:**

This study aimed to explore the willingness and attitudes of undergraduate nursing students enrolled in a geriatric nursing minor program toward pursuing careers in long-term care facilities.

**Methods:**

A qualitative phenomenological approach was adopted. Semi-structured, in-depth individual interviews were conducted with fourteen students minoring in geriatric nursing. Interview transcripts were analyzed using thematic analysis.

**Results:**

The analysis identified three primary themes: (1) Nascent Interest in Geriatric Care; (2) Perceived Deficiencies in Professional Competence for Geriatric Care; and (3) The “Government-Society-Family” Collaborative Geriatric Care Ecosystem Remains Underdeveloped.

**Conclusion:**

While nursing undergraduates recognize strong market demand for geriatric care professionals, their current career preferences favor hospital settings. This inclination is influenced by considerations of the social status associated with long-term care facilities, perceived misalignment between their skills and role requirements, limited career advancement pathways, and high labor intensity in long-term care.

## Introduction

The global acceleration of population aging has positioned elder care as a critical societal priority. According to the United Nations World Population Prospects 2024, the population aged 65 and over is projected to exceed 2.2 billion by the late 2070s, surpassing the number of children under age 18. By the mid-2030s, individuals aged 80 and above will reach 265 million, exceeding the infant population [1]. China has long been an aging society. Data from the Ministry of Civil Affairs indicate that by the end of 2023, the population aged 65 and above reached 216.76 million, accounting for 15.4% of the total population, with an old-age dependency ratio of 22.5% [2]. The old-age dependency ratio is defined as the number of people aged older than 65 years divided by number of working-age people aged 18–64 years, it is desirable that this figure remains low in any population [3].

Relative to younger populations, older adults experience progressive physiological decline and a high prevalence of multimorbidity. In China, approximately 72.68% of older adults suffer from two or more chronic conditions. The National Health Commission reports that around 190 million older individuals live with chronic diseases, and over 40 million require care due to disability—a figure projected to surpass 77 million by 2030. Furthermore, China has more than 9.5 million older adults with dementia, a number expected to exceed 40 million by 2050. Increasing life expectancy, combined with high rates of chronic disease, disability, and dementia among the oldest-old, has substantially increased the complexity of care needs.

Demographic shifts in family structure have further strained traditional care arrangements. China’s current working-age population is predominantly composed of individuals born during the era of the one-child policy [4]. Smaller household sizes have eroded the traditional reliance on familial support, leaving many families—especially those in rural areas without pension income—unable to meet the financial, temporal, and caregiving demands of supporting four to eight elderly parents and grandparents [5]. In this context, long-term care facilities have become a critical component of China’s elder-care system. By the end of 2023, China had 404,000 such facilities providing 8.23 million beds [2], increasingly serving as the preferred option for older adults with advanced age, chronic conditions, disability, or dementia, and their families [6,7].

Nevertheless, a severe shortage of professionally trained care personnel persists in long-term care facilities worldwide. Japan, facing rapid aging, will need 2.5 million geriatric care workers by 2025, with an estimated deficit of 340,000 [8]. Similar trends are seen in Western countries. The UK will require an additional 160,000 care workers by 2032 [9], and the US will need a substantial workforce to care for nearly 15 million older adults by 2050 [10]. In Germany, data from the Federal Statistical Office show that of approximately 1.7 million nursing care personnel, nearly 30% work in long-term care settings, and as demand for elderly care grows, the qualified personnel shortage intensifies [11,12].

Staffing in Chinese long-term care facilities typically consists of registered nurses and care aides who often receive only brief training before employment. Low social recognition, weak professional identity, high workloads, and persistent ageism contribute to high turnover, an aging workforce, and inadequate clinical competencies among care teams [13,14]. These staffing challenges compromise care quality and limit the capacity to meet residents’ needs. Research indicates that understaffing is associated with unfinished care, elevated rates of adverse events such as pressure injuries, deep-vein thrombosis, falls, depression, and increased mortality [15]. A systematic review further concludes that higher staffing levels and richer skill mixes in long-term care are correlated with better care outcomes, with registered nurse staffing inversely associated with pressure ulcers, hospitalizations, and urinary tract infections [16].

Developing a skilled workforce for elder care is therefore essential for China to respond actively to population aging, enhance older adults’ quality of life and social well-being, and promote the healthy development of the long-term care industry. In response, China has implemented reforms in geriatric care education, including the establishment of higher-education programs in elder services and management, and the promotion of professional certification schemes led by bodies such as the China Association of Social Welfare and relevant government departments [17,18].

To systematically examine the factors influencing nursing undergraduates’ willingness to work in long-term care, this study adopts the Social Cognitive Career Theory (SCCT) as its analytical framework [19]. SCCT posits that career interests, choices, and performance are shaped by the dynamic interaction of three core cognitive variables, namely self efficacy beliefs, outcome expectations, and personal goals, all of which are moderated by contextual factors. Self efficacy refers to an individual’s judgment of their ability to successfully perform career related tasks. Outcome expectations refer to an individual’s estimates of the physical, social, and self evaluative consequences of engaging in a particular activity. Personal goals translate interests into specific choice behaviors. Contextual factors, such as family support, policy environment, and social norms, indirectly influence the formation and adjustment of these cognitive variables by providing support or creating barriers. This theory provides an integrative analytical lens for understanding the interaction between individual cognition and environmental context.

Undergraduate nursing students represent a vital talent pool for improving the quality of elder care services. Their willingness to work in long-term care facilities significantly influences the future development of a high-quality geriatric care workforce [20,21]. Aligned with national policy, our university offers a minor in geriatric nursing and maintains collaborative partnerships with long-term care facilities, implementing a school-enterprise cooperative model to cultivate skilled care professionals [22,23].

This study examines the willingness of undergraduate nursing students who have voluntarily enrolled in a geriatric nursing minor to pursue careers in long-term care facilities, and explores the factors shaping their attitudes within the current sociopolitical context. The findings aim to provide insights for nursing educators and policymakers engaged in geriatric care workforce development.

## Participants and methods

### Participants

This qualitative study utilized a purposive sampling approach to recruit undergraduate nursing students enrolled in a geriatric nursing minor program, which operates as a cooperative “order-based” training partnership between our university and municipal long-term care facilities. Recruitment and data collection took place between September 2024 and June 2025. The minor curriculum was delivered during the fall semester of the participants’ junior academic year. A total of thirty and twenty-eight nursing undergraduates from the 2021 and 2022 admission cohorts, respectively, elected to pursue geriatric nursing as their minor specialization. Recruitment continued until thematic saturation was achieved, defined as the point at which subsequent interviews yielded no new substantive insights. Inclusion criteria consisted of: (1) voluntary enrollment in the geriatric nursing minor program; (2) participation in the university–facilities cooperative training initiative; (3) completion of all required theoretical and clinical coursework in geriatric nursing; and (4) provision of informed consent for participation and audio recording. Exclusion criteria included: (1) attendance below 90% in theoretical geriatric nursing courses, or (2) attendance below 100% in long-term care facilities practicum courses. A total of 14 students participated in the study. A total of 43 students met the inclusion and exclusion criteria. After 12 interviews, it was observed that no new themes or substantive elaborations on existing themes were emerging. To confirm saturation, two additional interviews were conducted, which likewise contributed no novel information. Consequently, a final sample of 14 undergraduate nursing students specializing in geriatric care participated in the study. As a token of appreciation for their time and contribution, each participant received one entry into a post-study lottery. Prizes consisted of practical items such as daily necessities, supermarket vouchers, and USB flash drives. All incentives were funded through the designated budget of the research team.

### Methods

#### Researcher characteristics.

***Research Team Members*.** The research team comprised four members. The first author, a university faculty member with a master’s degree, was responsible for laboratory management and practical course instruction for third- and fourth-year students. She possessed comprehensive knowledge of the students’ academic and clinical curriculum and was directly involved in their laboratory teaching and management. In this study, she was responsible for study design, conducting all 14 core in-depth interviews, managing audio recordings, writing field notes, transcript verification, independent coding, and manuscript writing.

The second author, a second-year master’s student in nursing, was responsible for participant recruitment, literature review, transcript verification, independent coding, and thematic framework discussion. The third author, a university faculty member with a master’s degree, was responsible for laboratory management and practical course instruction for first- and second-year students in fundamental nursing courses, with nine years of teaching and geriatric nursing research experience. She was responsible for study design review, coding arbitration, and educational practice validation. The fourth author held a master’s degree in nursing and was a head nurse at a tertiary hospital in Beijing, with eight years of geriatric care research and clinical management experience. She was responsible for clinical relevance verification, thematic framework review, and long-term care setting validation.

All team members had nursing professional backgrounds and received qualitative research training. The first, third, and fourth authors completed dedicated training in qualitative research methods and in-depth interview techniques during their respective master’s degree studies. The second author received qualitative research training during undergraduate studies and ongoing master’s coursework. Only the first author had a teaching relationship with participants.

***Positionality statement.*** The first author’s dual role as a faculty member and researcher may create several biases. First, power imbalance may lead participants to worry about academic evaluations, causing them to express overly positive career intentions or avoid sensitive topics like teaching evaluations. Second, relationship closeness may affect data quality. Familiar students might share more deeply due to trust, but could also participate just to save face. Unfamiliar students might feel nervous, avoidant, or give perfunctory responses. To mitigate these biases, we emphasized voluntary participation and grade independence in the consent form, used peer data collectors for supplementary conversations, conducted core interviews in neutral and private settings, had participants transcribe their own interviews, and maintained independent coding with team discussion of discrepancies.

***Mitigation of interviewer bias.*** To minimize power-related bias from the interviewer’s dual role as instructor and researcher, the following strategies were implemented.

Data Collection Phase: (1) Recruitment. Conducted by the second author; the first author was not involved. Recruitment occurred after class meetings when the instructor had left, ensuring voluntary participation. (2) Timing. All interviews were scheduled after grades were finalized. Fourth-year students were interviewed in mid-May after graduation exam scores were entered; third-year students in early April after final grades were submitted, before clinical internships. (3) Power dynamics. Participants received written assurances that content was for research only, that the interviewer had no role in academic evaluation, and that all information was confidential. The consent form provided Ethics Committee and university complaint channels. The interviewer maintained neutrality, avoided judgment, and refrained from leading questions. (4) Peer supplementary conversations. Two trained peer data collectors conducted informal conversations on sensitive topics. These data were used solely for validation, not core thematic analysis.

Data Analysis Phase: (1) Independent coding. The first and second authors independently coded all 14 transcripts, discussed disagreements, reviewed original context, and reached consensus. The third author arbitrated unresolved codes; the fourth author reviewed the framework for clinical and long-term care relevance. (2) Member checking. Finalized transcripts were returned to participants for verification. (3) Reflexive practice. The interviewer wrote field notes within 24 hours, documenting emotional reactions, value judgments, and power dynamic perceptions. The team reviewed these weekly to identify interpretive bias.

#### Interview guide development.

A semi-structured interview guide was developed based on the study objectives. The guide was piloted with two eligible students and refined iteratively to enhance clarity and relevance. The final guide included the following core domains: (1) What motivated your choice to pursue the geriatric nursing track? (2) How important is the role of nurses in long-term care facility? Please describe their potential contributions. (3) Would you consider working as a nurse in long-term care facilities in the future? Please explain your reasons. (4) Do you believe most nursing undergraduates are willing to work in long-term care facilities? Why or why not? (5) What are the strengths and weaknesses of the current educational collaboration between the university and partner long-term care facilities? (6) What improvements could be made to attract more young nursing graduates to geriatric care?

#### Data collection.

***Core Interviews (n = 14).*** Data were collected through face-to-face, in-depth, semi-structured individual interviews. Interviews were conducted in quiet, private study rooms to ensure confidentiality and minimize distractions. Only the interviewer and participant were present. Sessions were scheduled at times convenient for participants, avoiding meal breaks and rest periods. With prior consent, all interviews were audio-recorded. The interviewer also maintained handwritten field notes to document nonverbal cues and contextual observations. A neutral, non-judgmental stance was maintained throughout, and leading questions were avoided. Each interview lasted approximately 30–40 minutes.

***Peer supplementary conversations (n = 9).*** To obtain candid feedback on sensitive topics (e.g., educational collaboration), a peer supplementary conversation mechanism was introduced. Two class officers (a class monitor and an academic representative) served as peer data collectors. In the Chinese higher education system, a class monitor assists with daily class management and monitors student well-being, while an academic representative serves as a liaison between students and course instructors regarding learning needs and difficulties.

The peer data collectors were in the same class as participants during the first two years. After third-year specialization, they shared core courses but had different minor tracks (operating room and critical care, respectively), thus not meeting the inclusion criteria. Their role was limited to data collection and transcription; they did not participate in coding or thematic analysis.

Before the conversations, researchers provided 3.5 hours of training on: (1) reviewing of qualitative research methods (1h); (2) ethics and confidentiality, including non-disclosure to faculty (1h); (3) non-leading questioning and audio equipment (0.5h); and (4) role-playing exercises (1h). Both signed ethical commitment forms after training.

The two peer data collectors conducted informal, open-ended conversations with nine additional third-year students (not among the 14 core participants) in informal settings (e.g., cafeterias, playgrounds). These exploratory dialogues used prompts such as “What do you think about the teaching collaboration?” and “What are the strengths and weaknesses of our current training model?”

Prior to each session, participants received a full explanation of the study’s purpose, were assured of anonymity and confidentiality, and provided written informed consent. They were also informed of their right to withdraw at any time without consequence. The study adhered to the Consolidated Criteria for Reporting Qualitative Research (COREQ) to ensure transparency in data collection and reporting [24].

#### Data analysis method.

***Core data analysis (n = 14).*** Data collection and analysis occurred concurrently to allow for iterative refinement. Within 24 hours following each interview, a copy of the audio recording was provided to the research subject for verbatim transcription. The interviewer subsequently cross-referenced each transcript with observational notes taken during the interview to enhance data reliability.

Thematic analysis following the framework established by Braun and Clarke was applied to analyze the data [25]. Research team members independently coded the transcripts to identify salient statements and recurrent textual patterns, subsequently synthesizing the coded material. Divergences in thematic interpretation were resolved through structured team deliberation, comparative analysis, and iterative review until consensus on the final thematic framework was reached. Participant identifiers were replaced with anonymized codes throughout the analysis to ensure confidentiality.

***Supplementary data processing (n = 9).*** The nine supplementary audio recordings were transcribed by two class officers (a class monitor and an academic representative). Supplementary participants were identified by anonymized codes (S1–S9). The second author then cross-checked all supplementary transcripts against the original audio recordings to ensure accuracy. After verification, the peer data collectors returned the transcripts to the nine supplementary participants to confirm that the written records accurately reflected their intended meanings. Supplementary data were not subjected to thematic analysis. They were used solely for triangulation and validation of the“school teaching” subtheme.

#### Rigor and trustworthiness.

To ensure the credibility and transferability of findings, several strategies were employed based on Lincoln and Guba’s trustworthiness framework [26]. Participants were purposively selected to represent diversity in academic performance, family socioeconomic status, and experience with elder care. Rapport was established prior to interviews to foster open communication. Interview duration and probing were adapted flexibly to elicit rich data. The researcher maintained reflexivity by avoiding assumptions and attentively observing paraverbal and nonverbal cues. Transcripts were returned to participants for verification to confirm that the interpretations reflected their intended meanings.

#### Ethical considerations.

This study received ethical approval from the Ethics Committee of the School of Nursing (WNMC20250103). Given the privacy implications for interviewees, the following ethical measures were implemented: (1) Prior to interviews, participants were informed of the study’s purpose, methodology, and significance, with assurances that participant identities and information would remain confidential (2) Participants were notified that interviews would be audio-recorded, with consent obtained before recording commenced; (3) Participants were informed of their right to withdraw from the interview at any time.

## Results

### Demographic information of interviewees

A total of 14 undergraduate nursing students meeting the inclusion criteria participated in this study, comprising 5 fourth-year students undergoing clinical placement and 9 third-year students. Participants’ ages ranged from 20 to 22 years. Detailed demographic characteristics are presented in Table 1.

**Table 1:** Basic Information of Nursing Undergraduates with a Minor in Geriatric Nursing.

Case	Age (years)	Gender	Grade	Annual Household Income (CNY 10,000/year)	Registered Residence	Number of Elderly Family Members	Expected Salary (CNY/month)
**N1**	20-22	Female	Junior	10	Rural	4	7,000
**N2**	20-22	Female	Senior	14	City	3	10,000
**N3**	20-22	Female	Junior	16	City	1	7000
**N4**	20-22	Female	Junior	7	Rural	4	7000
**N5**	20-22	Female	Senior	6	Rural	2	8000
**N6**	20-22	Female	Junior	9	Rural	3	5000
**N7**	20-22	Female	Junior	11	City	4	7000
**N8**	20-22	Female	Senior	8	Rural	2	6000
**N9**	20-22	Female	Junior	11	Rural	1	8,000
**N10**	20-22	Female	Senior	15	City	4	10,000
**N11**	20-22	Female	Junior	10	Rural	4	7,000
**N12**	20-22	Female	Junior	9	Rural	3	7,000
**N13**	20-22	Female	Junior	5	Rural	2	5000
**N14**	20-22	Female	Senior	13	Rural	4	9,000

### Interview findings

#### Theme 1: Nascent interest in geriatric care.

***Influence of Traditional “Filial Piety” Values.*** Traditional Chinese culture emphasizes reverence and care for the elderly, a principle that has evolved from a familial ethic into a state-governed system over centuries. Since the Qin and Han dynasties, “filial piety” has been codified into law, with disrespect toward elders classified among the “Ten Major Crimes” [27]. This dual reinforcement through morality and law has deeply ingrained traditional filial piety in societal consciousness. These values positively shape nursing students’ career intentions toward geriatric care. As participants noted:

***N1:*** *“My grandmother raised me, but she passed away when I was in first grade. I couldn’t be there for her in her old age—a lifelong regret. From that moment, I resolved to dedicate myself to supporting more elderly people.”*

***N3:*** *“My grandfather once lived in a long-term care facility for a period. The caregivers there provided attentive care, and his life there was richer than at home. The humanistic care they offered during his treatment warmed my heart, and I want to pass on that warmth.”*

***N5:*** *“The elderly have dedicated their entire lives to societal development. When they can no longer care for themselves, we younger generation have a duty to provide them with quality services, passing love down through the generations. Only then can we expect to be well cared for in our own old age, allowing us to peacefully navigate life’s final journey.”*

These narratives reveal filial piety as a multidimensional moral-emotional anchor, transforming unresolved loss into redemptive purpose (N1), witnessed care quality into empathic commitment (N3), and cultural intergenerational responsibility into reciprocal obligation (N5). These pathways converge into positive social outcome expectations within the SCCT framework, operating through anticipated emotional fulfillment rather than instrumental calculation. This affective mode of expectation formation specifies how cultural values become internalized as career motives in collectivist settings, and suggests that geriatric care outcome expectations may carry stronger moral weight than in conventional domains.

***The critical role of nursing in an aging society.*** As societies age, the prevalence of multimorbidity, functional disability, dementia, and advanced age increases. In this context, the basic daily assistance traditionally provided by care aides is no longer sufficient. The involvement of professionally trained nurses has become a societal necessity. ***N10****: As the saying goes, “Treatment accounts for 30%, nursing for 70%.” Institutionalized seniors primarily rely on nursing for their physical well-being. Our four years of theoretical and practical training equip us not only to standardize daily care procedures but also to provide specialized nursing support in disease recovery and psychological care.*

***N11:*** *Nurses deliver specialized care for elderly illnesses while serving as vital bridges between residents, families, and doctors. Though these communication roles may seem small, they are fundamental to helping seniors adapt swiftly and preserving their dignity.*

These narratives reveal how students generate positive outcome expectations through professional identity. N10 emphasizes technical indispensability with the adage “Treatment accounts for 30%, nursing for 70%,” while N11 highlights relational coordination as a “communication bridge.” Together, they reframe geriatric nursing as skilled practice requiring professional training. This specifies a second source of outcome expectation formation alongside the emotion-driven pathway: students derive expectations from anticipated professional recognition and competence utilization.

***Perceived promising future of geriatric care.*** Given the pronounced trend of population aging, the demand for nursing professionals in long-term care is rising steadily. Participants viewed market needs as a strong indicator of the field’s potential.

***N4*** *: I don’t have a particular passion for geriatrics, but with hospitals hiring fewer nurses, I believe elderly care will be in high demand in the future. I chose this minor in elderly care partly to gain more knowledge, and partly to have a backup plan in case I can’t find a suitable hospital in the future.*

***N7:*** *Beyond our parents’ generation holding strong beliefs in “raising children for old age,” our generation of only children doesn’t expect our children to care for us. When we become frail, we’ll likely turn to long-term care facilities. Therefore, the future will require a large number of caregivers.*

Even students without a strong personal passion for geriatric care are attracted by the considerable market demand and employment prospects. N4 frames geriatric care as a “backup plan” amid hospital hiring contraction, while N7 projects demographic inevitability onto personal future planning. These pragmatic considerations form a third source of positive outcome expectations, distinct from moral motivations and professional identity. Like those earlier sources, however, this market-informed interest only shapes students’ initial interest, remaining at the cognitive level and failing to translate into goal commitment when confronted with efficacy deficits and environmental barriers.

#### Theme 2: Perceived deficiencies in professional competence for geriatric care.

***The imperative of compassion and patience.*** Long-term care facilities primarily serve older adults with advanced age, functional disabilities, or cognitive impairment. The gradual decline in physical function, coupled with deeply ingrained mindsets shaped by their life experiences, often results in communication difficulties. Participants emphasized that providing effective care in this context demands substantial compassion and patience.

***N3:*** *Older adults often have fixed mindsets. Coming from different generations, communicating with them can be very challenging. Before my grandfather entered long-term care facilities, he had dementia and a difficult personality. My family couldn’t manage his care at home. Caring for residents like him requires caregivers to have immense patience and compassion; otherwise, they can easily become emotionally depleted over time.*

***N6:*** *Caring for residents in long-term care facilities is different from treating patients in a hospital. It involves less acute medical intervention but demands greater compassion, patience, and personal responsibili*t*y. While a hospital is a temporary treatment site, a long-term care facility becomes their final home. Witnessing their emotional struggles makes me doubt my own ability to provide adequate emotional support.*

***N8*** *: During my long-term care facility practicum, I found communicating with residents quite challenging. I often had to speak very loudly and repeat myself multiple times for them to understand. This aspect of the work is difficult, and I don’t think someone without considerable patience could sustain it.*

These narratives converge on a critical insight: students perceive emotional labor not merely as a professional requirement but as a finite personal resource at risk of depletion. The convergence of N3’s exhaustion fear, N6’s self-doubt, and N8’s patience demands reveals low self-efficacy for emotional sustainability as the first dimension of Theme 2, undermining their confidence in pursuing geriatric care careers.

***Insufficiency of specialized geriatric knowledge.*** Nursing education in China primarily focuses on general medical knowledge, nursing theory, and clinical skills. However, older adults living in long-term care facilities face not only physical challenges but also psychological issues that require immediate attention upon admission.

***N10:*** *Although our university offer geriatric nursing minor, the curriculum remains largely disconnected from the actual needs of elderly residents in long term care facilities. The relatively relevant course “Geriatric Nursing” provides insufficient coverage of geriatric knowledge. The learning objective for many students, including myself, is merely to achieve a passing grade. I won’t need this knowledge if I work in a hospital or pursue graduate studies later. Unless I apply to a geriatric hospital or long-term care facility, last-minute exam cramming seems more useful than our current curriculum.*

***N12:*** *After interning at a long-term care facility, I realized we don’t truly understand the residents there. Even our instructors may have limited practical experience in this setting. Despite our geriatric care education, we still don’t know how to properly care for older adults living in long-term care facilities. Most residents suffer from multiple chronic conditions. Ensuring medication adherence is a major challenge; some even hide their pills. I believe we need to supplement our studies with geriatric care psychology to understand the underlying psychological needs behind their unusual behaviors.*

N10’s critique of curricular insufficiency and N12’s recognition of instructor-practice gaps expose a structural lag between classroom learning and institutional realities. This specifies the second dimension of Theme 2’s low self-efficacy: the perceived knowledge gap undermines students’ confidence that their acute-care training can address the multimorbidity and psychosocial complexity of long-term care.

***Need for an inquisitive and pioneering spirit.*** Participants perceived professional geriatric care in China as a field still in development, requiring caregivers who are not only knowledgeable but also willing to engage in exploration and knowledge creation to address existing gaps in practice and theory.

***N9:*** *Residents may spend their entire later years in long-term care facilities, even passing away there. Yet our textbooks offer minimal guidance on death education and end-of-life care—a critical component that should be standard practice for facility staff.*

***N11:*** *There are very few comprehensive books on geriatric care. I searched the library but couldn’t find systematic, in-depth knowledge. The existing knowledge we are currently learning is too limited to adequately address the care needs in elderly care facilities. The future requires not only knowledge disseminators but also knowledge creators. Care providers must cultivate intellectual curiosity to identify and solve emerging problems in geriatric practice, thereby providing higher-quality care. This represents a significant challenge for the profession.*

N9’s concern about the absence of death education and N11‘s critique of the lack of systematic geriatric knowledge reveal students’ aspiration to be “knowledge creators” rather than mere “knowledge users.” This perceived absence of a pioneering spirit adds a third dimension to Theme 2‘s low self-efficacy. Beyond concerns about emotional sustainability and specialized knowledge gaps, students feel unprepared to contribute to knowledge development in this emerging field, further undermining their confidence in pursuing long-term care careers.

#### Theme 3: The “government-society-family” collaborative geriatric care ecosystem remains underdeveloped.

***Inadequate policy safeguards for geriatric care professionals.*** Geriatric care is an emerging nursing specialty that has developed in response to population aging. Establishing a comprehensive support system that encompasses talent development, improved employment conditions, and structured career advancement is a protracted process. Participants highlighted that the current lack of robust, government-led policy frameworks directly impacts their career considerations, particularly regarding job stability and professional progression.

***N1:*** *From what I understand, long-term care facility positions offer low pay and unstable work with no sense of security. If the long-term care facility I work at closes down, I’d have to find a new job. At my age, it’s uncertain whether I could find suitable employment. So for high-risk long-term care facilities, I’d definitely prioritize stable hospital work.*

***N7:*** *Currently, long-term care facilities with higher wages are privately run and self-financed, meaning income is unstable. Government-run public long-term care facilities have low caregiver-to-resident ratios, offering stable but very low wages with heavy workloads. Neither option is easy, and hospitals pay better.*

***N13:*** *Working in a long-term care facility means a low income with no end in sight, no professional title advancement, and no promotions or raises. Other jobs at least offer room for advancement—if you work hard enough, you can earn more.*

N1’s fear of facility closure, N7‘s comparison of unstable private versus low-paid public facilities, and N13’s frustration with the absence of title advancement collectively reveal students‘ rational actor orientation. They weigh job stability, income security, and promotion potential as quantifiable variables in their career decisions. These policy level deficiencies constitute material barriers, the first dimension of Theme 3‘s environmental obstacles, directly discouraging students from committing to geriatric care careers regardless of their level of interest or outcome expectations.

***Persisting social stigma against geriatric care workers.*** Within China’s traditional social framework, caregiving labor has been historically gendered and socially constructed as “feminine” and “low-skilled” [28]. Geriatric care workers consequently face intersecting stigmas related to professional recognition, occupational identity, and social status, which impede their ability to gain deserved economic and social respect [29]. Participants expressed how this pervasive stigma personally affects their career decisions and social perceptions.

***N6:*** *I’m naturally sensitive and insecure. I worry about relatives and friends asking about my job if I become a care worker. I feel it would embarrass my parents.*

***N10:*** *Nurses have low social status but decent pay. Care workers have even lower status and poor income—it’s like stacking disadvantages. Even if I wanted to be a care worker, societal pressures heavily influence career choices.*

***N14:*** *Our neighbors, family, relatives, and friends are extremely competitive and love prying into private matters like income and occupation. Good news doesn’t travel far, but bad news spreads like wildfire. If I become a care worker, I’m terrified that acquaintances asking about my job will spread malicious rumors.*

N6’s fear of embarrassing parents, N10’s perception of “stacking disadvantages” from low status and poor income, and N14’s terror of malicious gossip reveal how stigma operates across interpersonal, familial, and community levels. This specifies the second dimension of environmental barriers in Theme 3: symbolic barriers, operating via “face” and reputation concerns in collectivist contexts, prove no less influential than material barriers in deterring career commitment.

***Insufficient family support for both supply and demand sides.*** China faces a severe shortage of geriatric care professionals, with most (50.5%) working 41–50 hours per week. Nearly half (48.9%) care for 10 or more residents daily, leading to immense work pressure and limited time for their own families [30]. Furthermore, some families of residents mistakenly equate paying fees with fulfilling filial piety after placing an elder in a facility. They may transfer core responsibilities of respect and care entirely onto professional staff, sometimes even imposing traditional expectations of“obedience”derived from filial piety culture.

***N2:*** *My parents don’t want me to become a care worker. It’s dirty and exhausting, but the main issue is the lack of respect. Few acknowledge the value of care workers.*

***N10:*** *I’m not married yet. Who would dare marry me after I become a caregiver? Low income, low status, and no time to care for my own elderly parents or children.*

***N8:*** *My parents hold quite traditional views. They believe it’s more important for women to find work that balances family responsibilities. After all, the idea that “men work outside the home while women manage the household” remains dominant. Women are still expected to find jobs that allow them to care for their families.*

N2’s parental disrespect concerns, N10’s marriageability anxiety, and N8’s traditional gender role pressures demonstrate family opposition as a bidirectional force. These family-level barriers deter entry into geriatric care while reinforcing hospital preference through concerns about wages, status, and work-family balance. This specifies the third dimension of environmental barriers in Theme 3: family-level constraints on social support compound the material and symbolic barriers identified previously, completing the multi-level ecosystem that channels talent away from long-term care.

## Discussion

### Social cognitive career theory as an integrative framework for understanding career decisions in long-term care

This study applies Social Cognitive Career Theory (SCCT) as an integrative framework [19]. SCCT posits that career choices are shaped by the dynamic interaction of three core cognitive variables, namely self-efficacy, outcome expectations, and personal goals, which are in turn moderated by external environmental factors [19]. Although this framework has been widely validated in Western individualistic contexts, its application in collectivist cultures and emerging industries remains insufficient.

The three themes identified in this study correspond systematically with the SCCT framework. Theme 1 reflects students’ outcome expectations, which operated across two dimensions: individual (market demand, employment prospects) and social (filial piety values, intergenerational responsibility). This bifurcated structure specifies the cultural manifestations of social outcome expectations in the Chinese context, wherein “family face” and “occupational prestige” constitute distinct considerations with independent explanatory power.

Theme 2 reveals low self-efficacy, and Theme 3 reflects environmental barriers. The critical finding is an interest–goal discontinuity: although students maintained positive outcome expectations and career interest, this interest failed to translate into personal goals and choice behavior due to low self-efficacy and environmental impediments. The majority of participants explicitly prioritized hospital employment, corroborating the obstruction of goal formation. This indicates that while outcome expectations can independently sustain interest, the translation of interest into goals requires the synergistic presence of self-efficacy and environmental support.

In summary, by applying SCCT, this study elucidates the underlying mechanism of nursing undergraduates’ career decision-making: their reluctance to enter long-term care is not merely a matter of individual preference, but rather an interest–goal discontinuity arising from the interplay of low self-efficacy, positive outcome expectations, and environmental barriers. This theoretical insight provides a foundation for understanding workforce shortages in geriatric care and designing multi-level interventions.

### Cultural values and market demand as catalysts for developing a high-quality geriatric care workforce

This study indicates that nursing undergraduates represent a potential pool of individuals interested in pursuing careers in elderly care, consistent with findings from Tao’s research [31]. Influenced by China’s millennia-old traditional culture, the traditional value of “filial piety” remains deeply ingrained among contemporary youth, framing respect and care for the elderly as both a moral virtue and a social responsibility. This cultural ethos serves as a significant motivational force for nursing students considering careers in geriatric care, especially in a context where care work is often socially devalued as “low-skilled” and gendered. Internalizing this sense of filial duty may help sustain professional commitment and resilience, providing a non-economic foundation for career retention [32]. Therefore, leveraging the cultural resonance of filial piety can help reframe geriatric care as a socially meaningful profession that honors both familial and societal obligations. This will establish profound ethical roots and cultural momentum for the development of elderly care talent.

Concurrently, China’s rapidly aging population, the declining capacity of family-based care, and rising expectations for quality of life in old age collectively generate substantial and growing demand for well-trained geriatric care professionals. This demand is not only quantitative but also qualitative, exerting external pressure to upgrade the training and professionalization of the workforce [33]. Market forces can incentivize structural improvements in talent development, such as competency-based grading and role differentiation, facilitating a shift from mere numerical expansion to quality-focused cultivation. Furthermore, a maturing market that recognizes and rewards high-quality services can support differentiated compensation and career pathways tied to skill level and performance. Such economic and professional incentives may help counteract the perception of geriatric care as “low-skill, low-pay” work and contribute to a virtuous cycle wherein demand drives quality improvement, quality enhances returns, and better returns attract and retain higher-caliber professionals.

### Comprehensive and systematic elder care curricula and teaching evaluation systems form the foundation for professional development in elder care talent

This study found that undergraduate nursing students perceive current geriatric care education as insufficient to address the increasingly complex and diverse needs within long-term care settings. Areas such as specialized dementia care, chronic disease management, rehabilitation support, and psychosocial intervention require professionals with broader knowledge bases, refined clinical competencies, and enhanced integrative skills. However, instructors in geriatric nursing programs often lack direct insight into the actual care needs and living contexts of older adults living in long-term care facilities. Additionally, the reliance on single-dimensional assessment methods fails to accurately quantify students’ learning outcomes. This is consistent with Huazhen Lu ‘s research results [34].

The evolution of care needs in residential facilities is reflected in the growing prominence of psychological and emotional well-being [35], which increasingly influences satisfaction among frail older adults [36]. Professional knowledge and skills grounded in real-world needs form the cornerstone of high-quality geriatric care. Current nursing education emphasizes nursing technical procedures over specialized competencies tailored to institutional care. A dedicated educational evaluation system for this setting has yet to be systematically developed.

Therefore, geriatric care education and training content must evolve with societal changes. Innovation in university-enterprise collaborative teaching models should be advanced. Geriatric care educators need to immerse themselves in care settings to understand the characteristics of residents and develop curricula and evaluation systems based on empirical needs. Educational frameworks should be multidisciplinary, integrating geriatric medicine, psychology, rehabilitation, nutrition, and social work, while strengthening students’ practical competencies and emergency response skills in real care environments. Furthermore, relevant courses should systematically incorporate contemporary interpretations of filial piety culture, blending it with geriatric ethics, communication training, and humanistic care education to cultivate professionals who are both clinically proficient and compassionately engaged.

### A supportive ecosystem as a prerequisite for sustainable development of geriatric care professionals

The findings of this study indicate that participants frequently cited income levels, social status, familial attitudes, and unclear career progression as factors influencing their career choices, suggesting that the broader professional ecosystem significantly shapes nursing students’ willingness to enter geriatric care, a conclusion supported by existing research [37]. Perceptions of geriatric care as an occupation with low prestige are often reinforced by the nature of the work, which involves intimate care, frequent exposure to illness and death, and a perceived role of “attendant” rather than professional. Such factors contribute to the stigmatization of the work as “dirty” or “low-status,” which can extend even to the families of caregivers [38]. Consequently, reshaping the social image of geriatric care workers is essential not only for enhancing the profession’s appeal but also for protecting the well-being of practitioners and supporting the sustainable development of the sector.

First, governmental leadership is essential in establishing clear professional standards and pathways. Alongside articulating workforce needs, the government should lead in developing and regularly updating national vocational standards, training curricula, and competency frameworks to ensure alignment with evolving industry demands. A nationally recognized skills grading and professional title system specific to geriatric care should be established to clarify career progression and mitigate perceived career ceilings. Furthermore, public funding should be strategically used to incentivize investment and provide foundational protections, supported by rigorous quality assessment and oversight mechanisms. Staffing ratios and qualification requirements should be integral to institutional accreditation, creating a “quality-driven” framework that encourages facilities to invest in human resource development.

Second, professional nursing associations and universities must collaboratively address China’s unique regulatory fragmentation. Unlike hospitals, which are uniformly regulated by the National Health Commission (NHC), long-term care facilities are managed by civil affairs authorities, while nurses are regulated by the NHC. This separation has resulted in a lack of systematic professional support for nursing in long-term care facilities, creating a regulatory vacuum described as “certification governed by health authorities, but personnel managed by civil affairs authorities.”

To bridge this gap, universities must update their curricula by establishing independent modules for long-term care nursing, incorporating institutional internships as mandatory clinical rotations with equal status to hospital internships, and ensuring that these courses receive recognition equivalent to hospital-based training in certification and career advancement pathways. Universities should also facilitate cross-sectoral dialogue among the NHC, hospital nursing leaders, facility managers, and civil affairs authorities to jointly develop dual-track competency standards that balance clinical skills and institutional care management capabilities.

Meanwhile, the Chinese Nursing Association should include representatives from long-term care facilities, grant facility managers a formal voice in standard-setting processes, and extend the annual hospital-based gerontological nurse certification to nurses working in long-term care facilities. Furthermore, it should promote a shift in social perceptions from “low-skill care” to “advanced specialty practice” through national academic conferences and community health education.

Third, media and public communication play a critical role in reshaping professional perceptions. Media and advocacy systems should actively contribute to constructing a contemporary, professional image of geriatric care workers. This involves highlighting both their core value of empowering healthy aging through professional skills and their unique blend of traditional filial piety ethics with modern dedication to their vocation. Such efforts will help dispel public misconceptions and reshape occupational identity. Empowering caregivers to articulate their professional value in public settings, including community seminars and public education initiatives, can also help demonstrate the professionalism, compassion, and social significance of geriatric care, gradually eroding the“low-skill, low-status”stigma.

Finally, family support must encompass both caregivers’ families (supply side) and care recipients’ families (demand side). Respect, understanding, and appreciation from recipients’ families serve as important non-financial incentives, enhancing caregivers’ sense of professional worth [39]. Simultaneously, active family involvement in care, whether through emotional support, decision-making, or supplementary assistance, can create a synergistic relationship with professional services, meeting both functional and emotional needs. From the caregiver’s family perspective, supportive attitudes are more likely to emerge when the profession is associated with fair compensation and social recognition. Thus, a positive social ecosystem is fundamental to securing stable familial support, which in turn provides a relational foundation for the career sustainability of geriatric care professionals.

## Conclusion

This study indicates that undergraduate nursing students, as a core talent reserve for the future of geriatric care, recognize the strong market demand and growth potential in this sector. However, their current career preferences remain inclined toward hospital settings, influenced by factors such as the relatively low social recognition of long-term care roles, perceived gaps between their training and the required competencies, limited clarity in career progression pathways, and concerns over high workloads in care facilities.

Therefore, to effectively cultivate a sustainable geriatric care workforce, policy and educational efforts should prioritize a dual strategy: (1) leveraging the cultural resonance of filial piety to enhance professional identity and commitment, and (2) aligning training with evolving societal needs through demand-driven curriculum design. It is essential to establish a coherent professional education and training system that responds proactively to the challenges of an aging population. Furthermore, a supportive ecosystem must be fostered through coordinated efforts among government, society, and families. Such collaboration can help create an enabling environment that attracts, retains, and develops skilled professionals.

This study makes two theoretical contributions to the literature. First, it extends SCCT to collectivist cultural contexts by elaborating the manifestations of social outcome expectations, identifying family ‘loss of face’ and occupational stigma as culturally specific social outcome expectations that shape career decisions.“ Second, this study supplements SCCT’s proposition on the joint effects of self-efficacy and outcome expectations, revealing a “decoupling” phenomenon in emerging industries: positive outcome expectations can sustain career interest despite low self-efficacy, yet fail to facilitate its translation into actual choice behavior.

## Supporting information

S1 Checklist Consolidated Criteria for Reporting Qualitative Research (COREQ). (PDF)
